# Comparative analysis of the recent publication trends in 4 representative journals in the spine field

**DOI:** 10.1097/MD.0000000000027716

**Published:** 2021-11-12

**Authors:** Kuhyun Yang, Hong-Gyu Baek, Dae-Chul Cho, Yoon Gyo Jung, Subum Lee, Jin Hoon Park

**Affiliations:** a Department of Neurosurgery, Gangneung Asan Hospital, University of Ulsan College of Medicine, Gangneung-si, Gangwon-do, Korea; b Department of Neurosurgery, School of Medicine, Kyungpook National University, Kyungpook National University Hospital, Daegu, Korea; c Department of Neurosurgery, Asan Medical Center, University of Ulsan College of Medicine, Seoul, Korea.

**Keywords:** *European Spinal Journal*, *Journal of Neurosurgery – Spine*, publication, *Spine*, *The Spine Journal*

## Abstract

We have analyzed and compared the publication trends in 4 representative spinal journals [*Spine, European Spinal Journal (EUS), The Spine Journal (TSJ),* and the *Journal of Neurosurgery – Spine (JNS spine)*] from 2016 to 2018.

A total of 3784 articles were published in the 4 representative journals: 1358, 1128, 685, and 613 articles in *Spine, EUS, TSJ,* and *JNS spine*, respectively. We compared and analyzed each periodical for the time taken (days) for the publication process, the distribution of specialties of the corresponding author, multicity of the investigative institutions, main disease entity, study type, and design.

The period from submission to online publication was 133, 216, 181, and 318 days in *Spine, EUS, TSJ,* and *JNS spine*, respectively. Corresponding authors with orthopedic specialties were more common in *Spine, EUS,* and *TSJ* than in *JNS spine*. Of particular note, corresponding authors who were neurosurgeons were the majority (55.8%) only in *JNS spine.* Single institution articles were by far the most common (average 92.8%) in all 4 journals. In all of the analyzed journals, the proportion of degenerative diseases was dominant with an average of 44.9%. The most frequent study type in all 4 journals was a clinical article (79.6, 72.1, 63.3, and 63.1%, respectively). In general, meta-analyses (average 4%) and randomized controlled comparative studies (average 5.2%) accounted for a very low percentage of the study types.

We believe that periodic analyses and comparisons of the characteristics of representative spine journals will help to shape the direction of future improvements.

## 1 Introduction

Numerous clinical and biomedical research articles on the human spine have been published recently and have greatly assisted spinal surgeons in their clinical practice and research activities.^[1,2]^ This rapid increase in the number of publications on the spine may be related to the increased numbers of spinal surgeries required in an aging society and unmet needs for knowledge due to rapid advances in new surgical techniques and instruments.^[3,4]^

Four well-established journals in the spinal field, *Spine, European Spinal Journal (EUS), The Spine Journal (TSJ),* and *Journal of neurosurgery – Spine (JNS spine)*, have been at the forefront of the recent clinical advances in various notable spinal disorders and treatments, and also in the expansion of basic research findings in this field through the use of animal studies or experimental trials. The 4 journals selected for this study were chosen after considering their history and impact factor rankings. The goal of this study was to check the recent publication trends by focusing on representative journals that literally any spine surgeon could aim to publish in at least once. That is why a relatively short, recent time frame was selected as the target period for analysis. However, by analyzing relatively short periods of these 4 journals, it is clear at the outset that there are limitations and difficulties in confirming all of the trends of spinal research worldwide.

Publication trends have developed for these representative journals over a period of many decades but there has been little analysis of this. We analyzed and compared the more recent publication trends for these periodicals from January 2016 to December 2018 as a way of providing useful feedback to spinal surgeons who are conducting existing research and preparing manuscripts for submission.

## 2 Methods

We analyzed a total of 180 issues of the selected journals (i.e., 72, 36, 36, and 36 [40%, 20%, 20%, and 20%] issues for *Spine, EUS, TSJ,* and *JNS spine*, respectively) containing 3784 articles (1358, 1128, 685, and 613 [35.9%, 29.8%, 18.1%, and 16.2%], respectively) that were published from January 2016 to December 2018. We directly checked all abstract and cover information of 3784 papers published in the 4 journals. The results were derived based on the raw data collected in a spreadsheet. This process was made possible by the dedicated contributions of the co-authors.

We compared and analyzed the following parameters: time taken (days) for the publication process, distribution of the specialties among the corresponding authors, the multicity nature of the participating institutions, the main disease entity, the study type, and study design. We analyzed the contributing countries/region & organizations for each of the 4 journals.

We noted the dates for submission, final acceptance and on-line publication and measured the intervals between these time points to rule out any biases related to the different times taken by the authors to revise their manuscript following a review. We found little influence of particular authors on the interval between the initial submission and final acceptance. We categorized the different specialties of the corresponding authors as follows: neurosurgeon, orthopedic surgeon, other specialty in the medical department (MD) (i.e., rehabilitation medicine, anesthesiology, etc) and non-MD. The multicity of institutions participating in each study was classed as single or multiple. The main disease entities were categorized as laboratory, trauma, tumor, deformity, degenerative lumbar, degenerative thoracic, degenerative cervical, or non-specific. The study types were grouped as follows: case report, experimental laboratory article, technical note, review article, and clinical study. The study design categories were laboratory study, randomized controlled comparative study (RCT), meta-analysis, retrospective cohort study, and prospective cohort study. In the categories related to the contribution analysis, the number and the ratio to the total were checked up to the top 5 ranking.

## 3 Results

**Figure 1:**
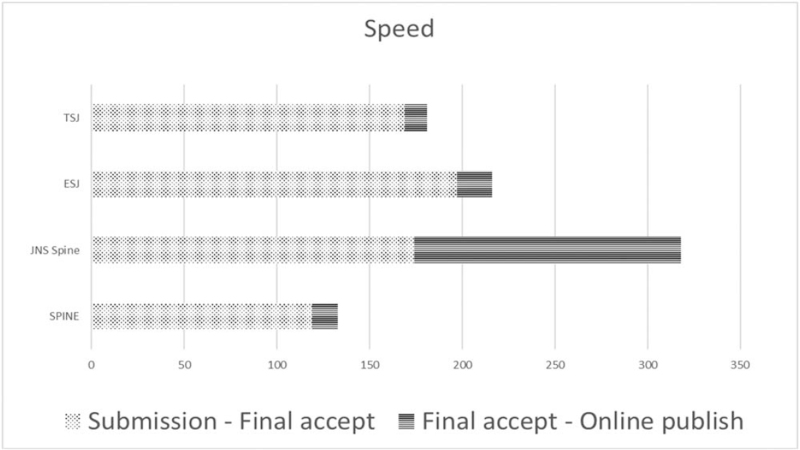
Time taken (days) for the publication process. The horizontal axis is the time taken to publication (days). *EUS* = *European Spinal Journal*, *JNS spine* = *Journal of Neurosurgery – Spine*, *TSJ* = *The Spine Journal*.

The periods from submission to acceptance were 119, 197, 169, and 174 days for *Spine, EUS, TSJ,* and *JNS spine*, respectively. The time from acceptance to online publication was the longest in *JNS spine* at 144 days compared to 14, 19, and 12 days for *Spine, EUS,* and *TSJ*, respectively. Hence, the times from submission to online publication were 133, 216, 181, and 318 days, respectively (Table 1 and Fig. 1).

**Table 1:** The comparative analysis for recent publication tendency of 4 representative spinal journals.

	*SPINE* (N = 1358)	*EUS* (N = 1128)	*TSJ* (N = 685)	*JNS spine* (N = 613)
Speed (d)				
Submission – online publish	133	216	181	318
Submission – accept	119	197	169	174
Accept – online publish	14	19	12	144
Author (%)				
OS	797 (58.7)	587 (52.0)	318 (46.4)	176 (28.7)
NS	180 (13.3)	140 (12.4)	92 (13.4)	342 (55.8)
MD	202 (14.9)	301 (26.7))	205 (29.9)	68 (11.1)
Non-MD	179 (13.2)	100 (8.9)	70 (10.2)	27 (4.4)
Institutional (%)				
Single	1238 (91.2)	1071 (94.9)	636 (92.8)	566 (92.3)
Multiple	120 (8.8)	57 (5.1)	49 (7.2)	47 (7.7)
Disease entity (%)				
Cervical degenerative	294 (23.5)	214 (19.0)	112 (16.4)	124 (20.1)
Thoracic degenerative	20 (1.0)	27 (2.5)	3 (0.4)	15 (2.4)
Lumbar degenerative	314 (25.4)	279 (24.7)	168 (24.5)	131 (21.3)
Deformity	332 (27.2)	300 (26.6)	78 (11.4)	65 (10.5)
Tumor	60 (4.8)	71 (6.3)	96 (14.0)	141 (23.0)
Trauma	36 (2.5)	76 (6.7)	31 (4.5)	42 (6.8)
Basic science	125 (15.6)	44 (3.8)	106 (15.5)	24 (3.9)
Bio-mechanic	69 (5.1)	45 (3.9)	38 (5.6)	34 (6.0)
Non-specific	108 (8.1)	72 (6.5)	53 (7.7)	37 (6.2)
Study type (%)				
Clinical article	1081 (79.6)	813 (72.1)	433 (63.3)	387 (63.1)
Review article	78 (5.7)	113 (10.0)	65 (9.5)	34 (5.5)
Technical note	3 (0.2)	31 (2.7)	28 (4.1)	13 (2.1)
Experimental article	159 (11.7)	129 (11.4)	125 (18.1)	79 (12.9)
Case report	37 (2.7)	42 (3.7)	34 (5.0)	100 (16.3)
Study design (%)				
Prospective	383 (28.2)	269 (23.8)	161 (23.5)	130 (21.2)
Retrospective	659 (48.5)	550 (48.8)	309 (45.1)	322 (52.4)
Meta-analysis	46 (3.5)	56 (5.1)	26 (3.8)	22 (3.6)
RCT	76 (5.7)	72 (6.2)	35 (5.1)	23 (3.8)
Laboratory story	194 (14.1)	181 (16.1)	154 (22.5)	116 (19.0)

Values are expressed as n (%). *EUS = European Spinal Journal*, *JNS spine* = *Journal of neurosurgery – Spine*, MD = medical degree, NS = neurosurgery, OS = orthopedic surgery, RCT = randomized controlled comparative study, *TSJ* = *The Spine Journal*.

**Figure 2:**
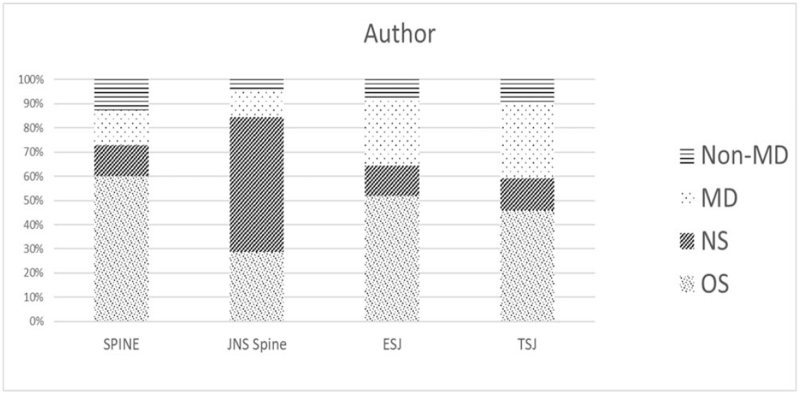
Distribution of the specialties practiced by the corresponding authors. A total of 1358, 1128, 685, and 613 articles published by *Spine*, *EUS, TSJ,* and *JNS spine* were analyzed for the specialty of the corresponding author. *EUS* = *European Spinal Journal*, *JNS spine* = *Journal of Neurosurgery – Spine*, NS = neurosurgery, MD = medical degree, OS = orthopedic surgery, *TSJ* = *The Spine Journal*.

There were 797, 587, 318, and 176 (58.7%, 52.0%, 46.4%, and 28.7%) orthopedic surgeons, respectively, listed as the corresponding author in *Spine, EUS, TSJ,* and *JNS spine*. This compared to 180, 140, 92, and 342 (13.3%, 12.4%, 13.4%, and 55.8%) neurosurgeons; 202, 301, 205, and 68 (14.9%, 26.7%, 29.9%, and 11.1%) non-neurosurgery and non-orthopedic MD specialties; 179, 100, 70, and 27 (13.2%, 8.9%, 10.2%, and 4.4%) non-MD, respectively. Notably, neurosurgeons were the most frequent corresponding author (55.8%) in *JNS spine* (Table 1 and Fig. 2).

**Figure 3:**
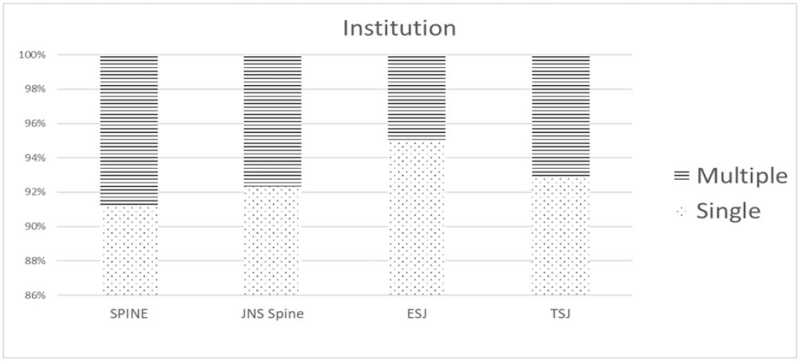
Multicity nature of the participating institutions. A total of 1358, 1128, 685, and 613 articles published by *Spine*, *EUS, TSJ,* and *JNS spine* were analyzed for the number of participating institutions. *EUS* = *European Spinal Journal*, *JNS spine* = *Journal of Neurosurgery – Spine*, *TSJ* = *The Spine Journal*.

There were 1238, 1071, 636, and 566 (91.2%, 94.9%, 92.8%, and 92.3%) single institution articles and 120, 57, 49, and 47 (8.8%, 5.1%, 7.2%, and 7.7%) multiple institution articles in *Spine, EUS, TSJ,* and *JNS spine* during the publication period under study. Single institution articles were therefore the substantial majority (average 92.8%) in all 4 journals (Table 1 and Fig. 3). The country that published the most papers in 3 journals, excluding *EUS*, was the United States. In *EUS*, China (260 articles, 23%) published more pieces than the USA (246 articles, 22%). As a single institution, the University of California system published many articles (Table 2).

**Table 2:** Ranking of contribution countries and organizations for 4 representative spinal journals.

	*SPINE* (N = 1358)	*EUS* (N = 1128)	*TSJ* (N = 685)	*JNS spine* (N = 613)
Contribution countries rank (N, %)				
1.	USA (690, 51)	China (260, 23)	USA (355, 49)	USA (443, 72)
2.	China (220, 16)	USA (246, 22)	China (73, 11)	Japan (70, 11)
3.	Japan (171, 12)	Japan (135, 12)	Canada (59, 9)	China (42, 7)
4.	Canada (97, 7)	Germ. (134, 12)	S. Korea (46, 7)	Canada (39, 6)
5.	S. Korea (68, 5)	France (116, 10)	Japan (45, 7)	Germ. (27, 4)
Contribution organizations (N)				
1.	Univ. of California sys. (79)	Univ. of California sys. (50)	Harvard Univ. (56)	Univ. of California sys. (61)
2.	Hosp. special surg. (65)	IRCCS Orthpedico Galeazzi (47)	Univ. of California sys. (42)	Univ. of Verginia (38)
3.	Johns Hopkins Univ. (53)	CHU Bordeaux (37)	Messachusetts gen. Hosp. (37)	Johns Hopkins Univ. (35)
4.	Harvard Univ. (51)	New York Univ. (28)	Hosp. special surg. (32)	Rush Univ. (30)
5.	Univ. of Verginia (49)	Schulthess clinic (27)	Brigham & Women's hosp. (31)	Brown Univ. (23)

*EUS = European Spinal Journal*, Germ. = Germany, Hosp. specific surg. = hospital special surgeon, *JNS spine* = *Journal of Neurosurgery – Spine*, Messachusetts gen. hosp. = Mesachusetts general hospital, N = number, S. Korea = South Korea, *TSJ = The Spine Journal*, Univ. of California sys. = University of California System, USA = United States of America.

**Figure 4:**
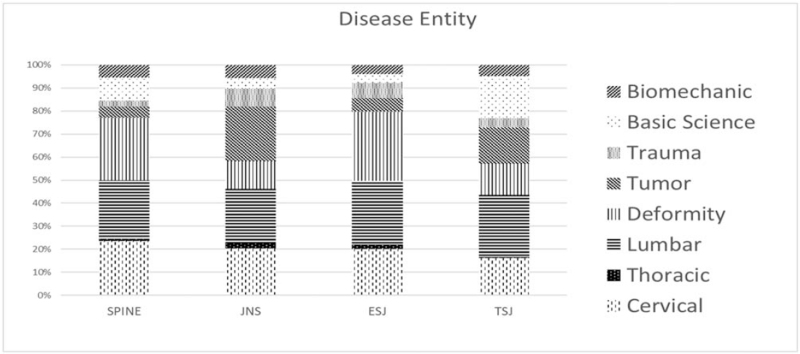
Distribution of the main spinal disease entities. A total of 1250, 1056, 632, and 576 articles were analyzed, excluding the non-specific disease entity group, from *Spine*, *EUS, TSJ,* and *JNS spine* for the main spinal disease entity covered by the study. *EUS* = *European Spinal Journal*, *JNS spine* = *Journal of Neurosurgery – Spine*, *TSJ* = *The Spine Journal*.

In terms of disease entities, a non-specific type was described in 108, 72, 53, and 37 (8.1%, 6.5%, 7.7%, and 6.1%) reports in *Spine, EUS, TSJ,* and *JNS spine*, respectively. For defined disorders of the spine, the descending order of frequency in the published papers was deformity, 332; lumbar, 314; and cervical degenerative disease, 294 (24.4%, 23.1%, and 21.6%) in *Spine*; deformity, 300; lumbar, 279; and cervical degenerative disease 214 (26.6%, 24.7%, and 19.0%) in *EUS*; lumbar, 168; cervical, 112; basic science, 106; and tumor, 96 (24.5%, 16.4%, 15.5%, and 14.0%) in *TSJ*; and tumor, 141; lumbar, 131; and cervical degenerative disease, 124 (23.0%, 21.2%, and 20.1%) in *JNS spine* (Table 1 and Fig. 4).

**Figure 5:**
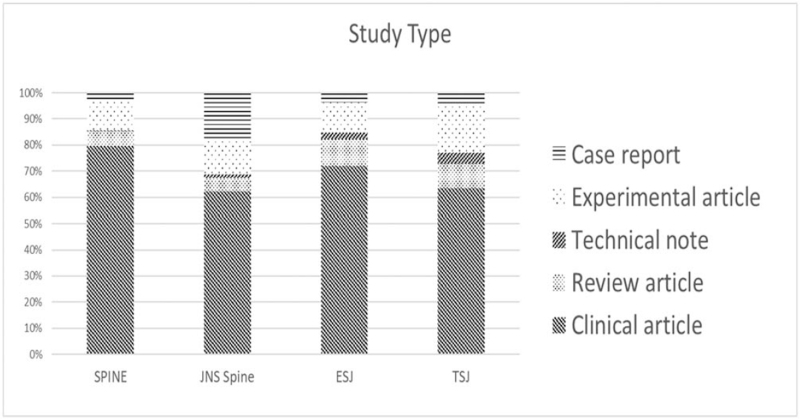
Distribution of the study types. A total of 1358, 1128, 685, and 613 articles published by *Spine*, *EUS, TSJ,* and *JNS spine* were analyzed for study type. *EUS* = *European Spinal Journal*, *JNS spine* = *Journal of Neurosurgery – Spine*, *TSJ* = *The Spine Journal*.

The most frequent study type in all 4 journals was a clinical article (79.6%, 72.1%, 63.3%, and 63.1%). Except for *JNS spine*, the second most frequent study type was an experimental article (i.e., 11.7%, 11.4%, and 18.1% in *Spine, EUS,* and *TSJ,* respectively). Case reports (16.3%) were the second most common study type in *JNS spine* (Table 1 and Fig. 5).

**Figure 6:**
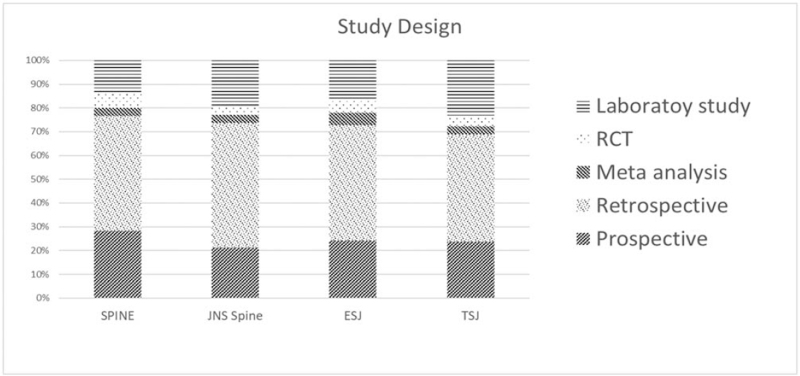
Distribution of the study designs. A total of 1358, 1128, 685, and 613 articles published by *Spine*, *EUS, TSJ,* and *JNS spine* were analyzed for study design. *EUS* = *European Spinal Journal*, *JNS spine* = *Journal of Neurosurgery – Spine*, *TSJ* = *The Spine Journal*.

In terms of the study design in the 4 journals under analysis, the overall trend was found to be similar. Retrospective studies were the most common (an average of 48.7%) without much variation, followed by prospective studies (24.2%). In general, meta-analyses and RCT studies accounted for a low percentage of the published papers. In the case of *EUS*, the laboratory study type was the most frequent with this journal having the lowest retrospective type study ratio among the 4 periodicals (Table 1 and Fig. 6).

**Figure 7:**
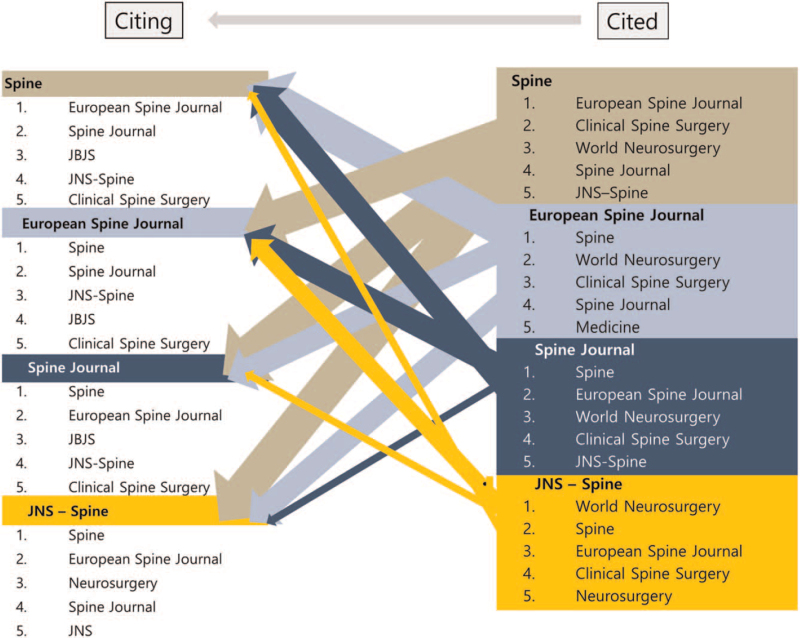
Proportion analysis of citing and cited journals. The ranking of the journals in which the 4 journals were cited and the ranking of the journals that were cited by the 4 journals were respectively shown up to 5th place. In the figure, the thickness of the arrow is proportional to the number cited. In this analysis, self-citing cases were excluded. The 4 journals: *EUS* = *European Spinal Journal*, *JNS spine* = *Journal of Neurosurgery – Spine*, *TSJ* = *The Spine Journal*.

The articles of 4 journals were analyzed to see which journals they cited and in which journals were the articles of the 4 journals cited. In this analysis, self-citing cases were excluded. Except for *Spine* (self-citing), the articles in the other 3 journals mostly cited articles in *Spine* (Fig. 7).

## 4 Discussion

We selected these 4 journals for the following reasons. Before 2001, spine research was published just by 2 journals, *Spine* and *EUS*.^[5]^ These 2 are spine journals with a long history (publication start year: *Spine*, 1976; *EUS*, 1992). *Spine* has the largest number of affiliated societies. The prominent spine societies, the Scoliosis Research Society and the Cervical Spine Research Society, are representative. It also has the most multinational affiliated societies such as the Argentine Society for the Study of Spine Pathology, Asia Pacific Orthopaedic Association, Chinese Orthopaedic Association, Finnish Spine Research Society, Japan Spine Research Society, Korean Society of Spine Surgery, Spine Section of the Hellenic Orthopaedic Association, and Spine Society of Australia. *EUS* is the official publication of the European Spine Society, European Spinal Deformity Society, and European Section of the Cervical Spine Research Society. *TSJ* is the official journal of the North American Spine Society, one of the most influential spine specialists today. *JNS* is the official journal of the American Association of Neurological Surgeons, which has been published since 1944, and has been isolated as *JNS spine* since July 2004. It has the highest influence among neurosurgery journals that only focus on the spine field.

The 5 journals (the 4 journals we studied and *Clinical Spine Surgery*) are the only spine specific publications indexed on MEDLINE.^[5]^ Among them, the top 4 journals were selected and investigated based on their impact factor (2018 journal impact factor: *TSJ*, 3.196; *JNS spine*, 2.998; *Spine*, 2.903; *EUS*, 2.513; *Clinical Spine Surgery*, 1.726). In the proportion analysis of citing and cited journals (Fig. 7), the articles in *Spine* mostly cited articles in *EUS*. The articles in *EUS* and *TSJ* mostly cited articles in *Spine*. The articles in *Spine* mostly cited articles in *EUS*. Interestingly, the articles in *JNS* s*pine* mostly cited articles in *World Neurosurgery,* with the exception of the 4 journals we analyzed, *Clinical Spine Surgery* ranked relatively well in most of the categories.

The principal reason for the longest time to publication in *JNS spine* appears to be the proofreading process. The intervals from submission to final acceptance were comparable among the 4 journals we analyzed. Notably, however, the interval from acceptance to on-line publication was nearly 10 times longer in *JNS spine* than in the other 3 journals. *Spine, EUS,* and *TSJ* progress their page proofing processes with PDF files. However, *JNS spine* has a two-step process that uses a Microsoft word file first and then a PDF, which may be the cause of the delay. *Spine* handled the highest number of articles and showed the fastest decision-making process.

Orthopedic surgeon was the most common specialty of the corresponding author in *Spine, EUS,* and *TSJ*. In these 3 journals, the most prominent discipline for the corresponding authors in descending order were OS, other MD, NS, and non-MD. Neurosurgeons were the primary contributors to *JNS spine* only. *Spine, TSJ,* and *EUS* are mainly handled by orthopedic surgeons and *JNS spine* by neurosurgeons. Rather than acceptance style, submission preference by authors to those 4 journals could have played such a role. Orthopedic surgeons might have preferred to submit to *EUS*, *Spine*, and *TSJ,* whereas neurosurgeons to *JNS spine*. This problem could be overcome with a questionnaire directed to spine surgeons.

In all 4 journals under evaluation, single-center studies were in the strong majority (92.8%). Due to the relatively low percentage of multicenter studies, meta-analysis was the least common type of study in all 4 journals. If it was difficult to define the disease entities for a given publication, such as letters to the editor for example, we classified them as non-specific. There was no significant difference found in the frequency of this non-specific category among the 4 journals. Degenerative spinal diseases including those at the cervical, thoracic, and lumbar levels were predominant across all 4 periodicals with an average of 44.9%. *Spine* and *EUS* had the second highest frequency of deformity groups among the degenerative disease categories. In *TSJ*, however, a higher proportion of the studies involved tumor cases (14.0%) and basic science (15.5%) compared to spinal deformity cases and this journal had the highest prevalence of basic science reports. *JNS spine* had the highest proportion of studies involving tumor patients (23.0%) among the 4 journals.

In our study type analysis, clinical articles predominated in all 4 journals with an average of 69.5%, which was an expected finding. *Spine, EUS,* and *TSJ* had the second highest proportion of experimental articles among this clinical report category with *TSJ* publishing the most experimental studies among the 4 journals. The proportion of case reports was the highest in *JNS spine* among the 4 journals (16.3%). The publication of case reports seems to have become rarer and in many ways they are more difficult to be accepted by the leading medical journals at present. This may be due to a reduced interest in this type of study or of single rare cases, possibly due to the ever increasing exchange of medical information through other means. We contend however that case reports still have great value for increasing our understanding of rare diseases and in novel treatment development. In the present age of rapidly developing and diverse medical technologies, a solid body of evidence and an exchange of views is important for any given disorder. The number of case reports published in *JNS spine* is not many, but we believe that the continuing acceptance of this type of study by this journal makes a beneficial contribution to the field.

Our study design analysis revealed that meta-analyses and RCTs are significantly less frequent types of reports in all 4 journals. As mentioned above, the overwhelmingly low ratio of multicenter to single-center studies may be the reason for the low number of meta-analysis studies. In the case of RCTs, the absolute number of subjects is just as important as a well-designed protocol for increasing the level of consensus in the evidence and generalizing the results of the study. As a general observation, the very few meta-analyses and RCT studies published by all 4 journals is worthy of discussion and should not be overlooked. The low numbers of published randomized studies are not due to low acceptance rates for those papers. Everybody knows that prospective studies are hard to conduct compared to retrospective studies and are scarce in number for this reason. If submissions related to such papers have been higher, the acceptance rate of those papers would have been higher than the one of retrospective studies.

We found that almost 50% of the articles in all 4 journals were dealing with degenerative spinal disease. This observation was not surprising as these types of spinal disease are globally very prevalent and are therefore treated in almost every hospital in every region of the world. Nevertheless, we contend that the remarkably low proportion of prospectively designed multicenter studies of spinal disorders utilizing meta-analytic approaches is regrettable, and that collaborative large-scale studies of this nature need to be actively encouraged.

The present study has limitations. First, the volume and number of papers are different for each journal. *Spine* publishes 24 issues a year, and the other 3 journals (*EUS, TSJ,* and *JNS spine*) publish 12 issues a year. Because we analyzed the publications based on the cross section of the period, the number of issues was not unified. Also, since the number of papers published for each issue in each journal is different, the number of papers could not be unified. Therefore, we analyzed the proportion of articles in each journal, not the number of articles. Second, the current study analyzed the papers published from 2016 to 2018. Therefore, it is unable to show the latest trends. Lastly, not only popularity could be a reason to select only 4 journals of spine research. It should be representative of all of the world. However, *Asian Spine Journal* is one of the oldest international peer-reviewed journals (publication start year: 2007) had not been included in the present study.

## 5 Conclusion

Surgeons, other clinicians, and researchers with a focus on the human spine will be very familiar with the reputation and prestige of *Spine, EUS, TSJ,* and *JNS spine*, 4 leading journals that have published some of the most important biomedical findings from the world's leading practitioners in this field. The authors believe that periodically analyzing and comparing the publication characteristics of these representative spine journals will help to shape the direction of future advancements in this field by providing insights into possible trends and biases in the types of studies that are being accepted.

## Author contributions

**Conceptualization:** Subum Lee, Jin Hoon Park.

**Data curation:** Yoon Gyo Jung, Subum Lee.

**Formal analysis:** Kuhyun Yang, Hong-Gyu Baek, Yoon Gyo Jung, Subum Lee, Jin Hoon Park.

**Investigation:** Subum Lee.

**Methodology:** Jin Hoon Park.

**Project administration:** Jin Hoon Park.

**Software:** Yoon Gyo Jung.

**Supervision:** Dae-Chul Cho, Jin Hoon Park.

**Validation:** Kuhyun Yang, Hong-Gyu Baek, Dae-Chul Cho.

**Visualization:** Subum Lee.

**Writing – original draft:** Kuhyun Yang.

**Writing – review & editing:** Kuhyun Yang, Hong-Gyu Baek, Dae-Chul Cho, Subum Lee, Jin Hoon Park.
